# Review of methods to derive the heartbeat-evoked potential: past practices and future directions

**DOI:** 10.1093/scan/nsag057

**Published:** 2026-09-17

**Authors:** Rania-Iman Virjee, Rohan Kandasamy, Sarah N Garfinkel, David W Carmichael, Mahinda Yogarajah

**Affiliations:** Institute of Cognitive Neuroscience, University College London, Alexandra House, 17-19 Queen Square, London, WC1N 3AZ, United Kingdom; Institute of Cognitive Neuroscience, University College London, Alexandra House, 17-19 Queen Square, London, WC1N 3AZ, United Kingdom; Department of Clinical Neurophysiology, The National Hospital for Neurology and Neurosurgery, Queen Square, London, WC1N 3BG, United Kingdom; The Queen Square Institute of Neurology, UCL, Queen Square, London, WC1N 3BG, United Kingdom; Institute of Cognitive Neuroscience, University College London, Alexandra House, 17-19 Queen Square, London, WC1N 3AZ, United Kingdom; School of Biomedical Engineering and Imaging Sciences, King’s College London, St. Thomas’ Hospital, Westminster Bridge Road, London, SE1 7EH, United Kingdom; Developmental Imaging and Biophysics, UCL Institute of Child Health, University College London, 30 Guilford St, London, WC1N 1EH, United Kingdom; Department of Clinical Neurophysiology, The National Hospital for Neurology and Neurosurgery, Queen Square, London, WC1N 3BG, United Kingdom; The Queen Square Institute of Neurology, UCL, Queen Square, London, WC1N 3BG, United Kingdom; The Epilepsy Society, Chalfont St Peter, Buckinghamshire, SL9 0RJ, United Kingdom

**Keywords:** Heartbeat-Evoked Potential (HEP), Interoception, Electroencephalogram (EEG), Magnetoencephalogram (MEG), Methodological approaches, Scoping review

## Abstract

The heartbeat-evoked potential (HEP) is an implicit, electrophysiological marker of cortical heartbeat processing and interoception, with increasing clinical relevance. However, on the scalp, HEP are low-amplitude signals mixed with cardiac field artefacts (CFA), requiring signal processing pipelines to separate HEP from CFA. This review evaluates current analytical approaches and addresses methodological gaps in HEP pipelines. A scoping review of the literature was conducted focusing on previously published HEP studies. This was to extract HEP processing methods and parameters used in EEG (199 included papers) and MEG (8 included papers). Parameters investigated included the HEP window, filters, baseline correction window, epoch timeframes, minimum RR intervals, epoch amplitude rejection threshold, and CFA removal. EEG and MEG studies revealed clear heterogeneity and variability in the methods used to derive HEP. These clear inconsistencies in HEP parameter use and reporting would appear to challenge the level replicability between studies. In order to ensure reproducibility and transparency, publications should report critical values for reliable HEP extraction, emphasizing the need for standardized methods to enhance study comparability and reproducibility.

## Introduction

Interoception is the process by which the nervous system senses, interprets, and integrates signals originating from within the body (e.g. hunger, thirst, cardiac and respiratory signals), providing a moment-by-moment mapping of the body’s internal landscape across conscious and unconscious levels (Khalsa *et al*. 2018). The heart continuously and cyclically communicates with the brain, impacting cognitive and emotional processes (Park and Blanke 2019). First investigated by Schandry *et al*. (1986) and Jones *et al*. (1986), the heartbeat-evoked potential (HEP) is a cortical brain response that is time-locked to each heartbeat, and which is consistently interpreted as an implicit, electrophysiological marker reflecting the cortical processing of heartbeats (Kumral *et al*. 2022), and more broadly interoception. Since initial publications in the early 1980s, HEP has gradually gained more attention in the literature, with publications rising steadily from 2015 onward and reaching a peak of 34 studies in 2025.

HEP appears to be a meaningful clinical measure (Suksasilp and Garfinkel 2022). For example, HEP has been shown to have trait variation between groups such as in the ageing population (Kamp *et al*. 2021), and in groups with psychiatric conditions characterized by emotion dysregulation, including borderline personality disorder (BPD) (Flasbeck *et al*. 2020), and generalized anxiety disorder (GAD) (Pang *et al*. 2019, Schmitz *et al*. 2021). Other studies have shown state differences in HEP, including relationships with interoceptive processing (Baranauskas *et al*. 2017, Hodossy *et al*. 2021, Desmedt *et al*. 2023), as well as in the periods preceding functional seizures (FS) (Elkommos *et al*. 2023, Kandasamy *et al*. 2026). Other studies aiming to validate brain stimulation techniques to interfere with interoceptive processes (Pollatos *et al*. 2016) and novel behavioural tasks to assess interoceptive processing in infants (Maister *et al*. 2017) have also utilized HEP, although these have been debated by the community (Coll *et al*. 2017).

Despite these promising earlier indicators of HEP utility, a fundamental inherent constraint is its susceptibility to contamination from direct electrical potential and associated magnetic flux when measuring it with electroencephalogram (EEG) or magnetoencephalography (MEG). This contamination arises from the electrophysiological activity of the heart which generates an electric field (i.e. the cardiac field artefact—CFA) that spreads throughout the whole body including the scalp (Arnau *et al*. 2023). Consequently, removing the CFA from HEP presents a significant challenge. This challenge stems not only from the limited capability to precisely measure the cardiac field and the incomplete understanding of its propagation towards the scalp (Yuan *et al*. 2007), but more fundamentally from the high temporal congruency between signal and artefact. The HEP is a small-amplitude transient neural response in the order of a couple of microvolts (approximately 1–5 µV), time-locked to the heartbeat—the same event that generates the CFA—which can be an order of magnitude larger (for example −10 to +10 µV) (Dirlich *et al*. 1997). As described by Dirlich *et al*. (1997), the CFA is largest during QRS complex and the T wave, exhibiting rapid temporal changes during the former and slower variations during the latter, directly overlapping with the HEP window. This temporal overlap inherently limits the effectiveness of conventional ERP-style subtraction methods. In addition, CFA should not be conceptualized as a static nuisance variable, but rather as a dynamic artefact where its amplitude and morphology vary systematically with posture, heart rate, respiratory phase, cardiovascular fluctuations and neck morphology of each individual (Dirlich *et al*. 1997, Kern *et al*. 2013). These are also the physiological measures that many HEP studies investigate, thus creating a systematic confound whereby observed HEP differences between groups and conditions may in part be driven by differences in CFA rather than true neural responses. Indeed, Buot *et al*. (2021) demonstrated that HEP amplitude co-fluctuates with stroke volume beat-to-beat, only in the absence of suitable CFA removal. This reveals how inadequate CFA correction directly introduces bias into HEP results, underscoring the need for methodological rigour and standardization in CFA removal for valid inference of HEP findings.

Studies have suggested various physiological sources underlying HEP (Park and Blanke 2019). Previous work suggests that the physiological pathways are multifaceted and complex. HEP components can be noted in multiple cortical regions and temporal intervals, which may relate to distinct afferent signals from the carotid arteries and baroreceptors at the aortic arch (Gray *et al*. 2007, Garfinkel and Critchley 2016), the cardiac afferent neurons (Tahsili-Fahadan and Geocadin 2017), somatosensory pathways (Khalsa *et al*. 2009, Kern *et al*. 2013) and cortical neuro-vascular coupling (Kim *et al*. 2016). To date, studies have reported HEP in numerous sensor locations: frontal (Canales-Johnson *et al*. 2015, Babo-Rebelo *et al*. 2016), central (Montoya *et al*. 1993, Petzschner *et al*. 2019) and parietal channels (Pollatos and Schandry 2004, Kern *et al*. 2013, Babo-Rebelo *et al*. 2019). This variability in HEP topography is thought to be generated by various methodological considerations and the widespread distribution of interoceptive processing in the brain including the insula, cingulate cortex, somatosensory cortex and amygdala. Variation in experimental design, such as the positioning of the reference, the number of electrodes, and the performance of the EEG amplifier can all in turn affect HEP topographies (Park and Blanke 2019). The absence of control conditions in some experimental designs also contributes to the complexity in understanding physiological pathways underlying HEP (Coll *et al*. 2017) As Coll *et al*. (2021) identified, a systematic meta-analysis of HEP research is essential to render the use of HEP as a reliable, powerful and well-grounded indicator of cortical interoceptive processing of cardiac signals and to establish consistent processing methodologies to use HEP in clinical and research settings (Coll *et al*. 2021, Suksasilp and Garfinkel 2022).

The aims of the review are to better understand HEP processing methods currently used in both EEG and MEG studies, and to explore and quantify variability in the methodology and parameters used to record the HEP. Finally, this review aims to take an initial step towards fostering consensus about optimal and standardized HEP processing methods.

## Materials and methods

### Scoping review search strategy

**Figure 1:**
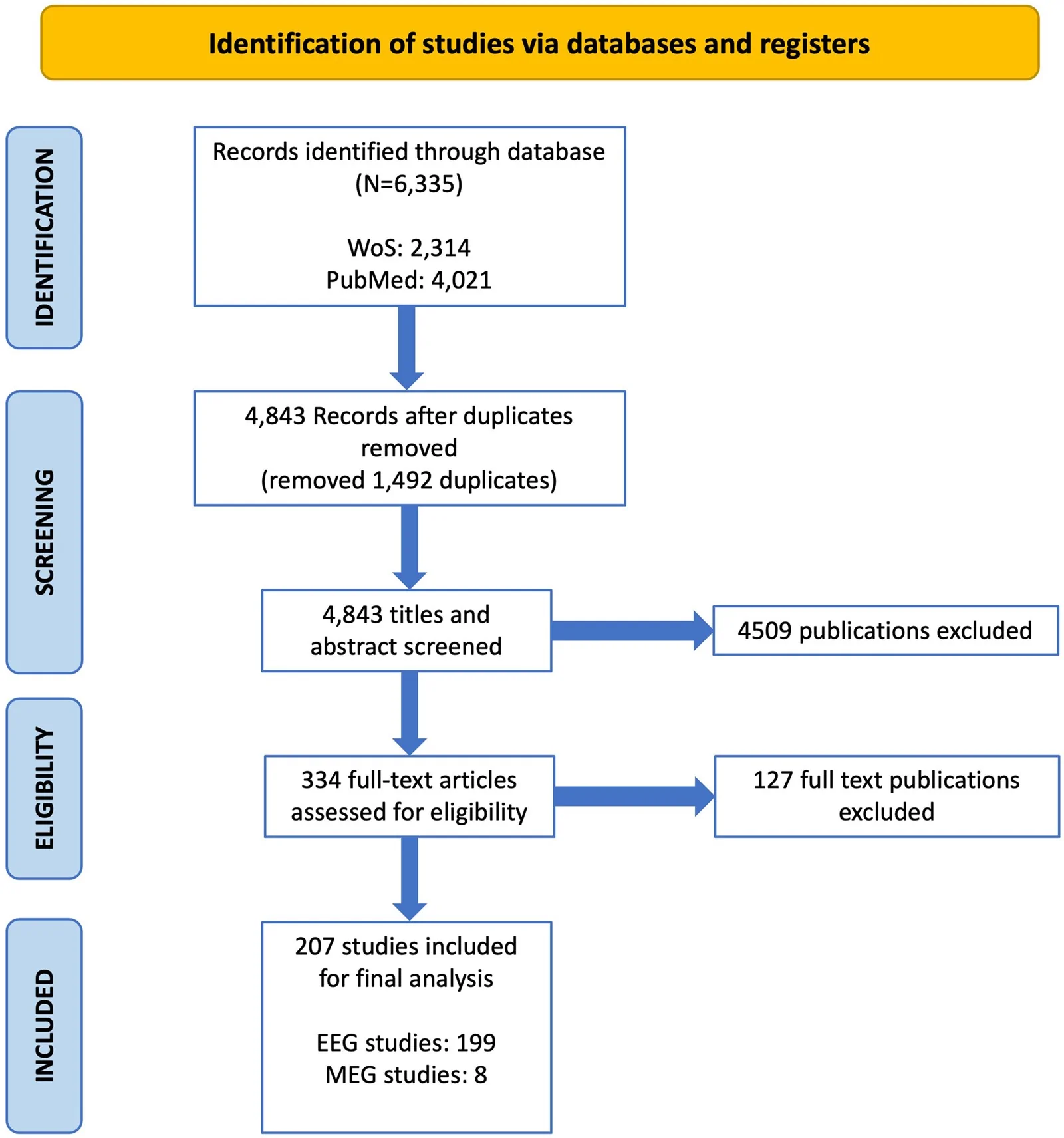
PRISMA flow diagram demonstrating the total number of publications included and removed at the different stages and the reasons for exclusion.

The literature search involved two electronic databases, Web of Science and PubMed, which yielded 6,335 potential eligible studies. This was reduced to 207 studies (EEG and MEG studies) after removal of duplicates and exclusion of studies (Fig. 1). This search spanned from 1952 to April 2026. A series of different combinations of key words were used for the literature search including Boolean operators (‘AND’ and ‘OR’) (Table 1). The full list of the studies included in the data extraction is provided as a supplementary file S1.

**Table 1:** Search strategies used in for PubMed and Web of Science. Search term blocks were combined as follows: (A and B) or (C and D).

Block	Concept	Search terms
**A**	Cardiac/interoceptive terms	(“heart” OR “heart rate” OR “heartbeat” OR “cardiac” OR “cardioception” OR “interoception”)
**B**	General evoked response terms	(“evoked potential” OR “evoked potentials” OR “evoked response” OR “evoked responses”)
**C**	HEP specific terms	(“heartbeat evoked potential” OR “heartbeat-evoked potential” OR “heartbeat evoked response” OR “heartbeat-evoked response” OR “heart evoked potential” OR “heart evoked response” OR “heart-evoked potential” OR “heart-evoked response” OR “cardiac evoked potential” OR “cardiac evoked response” OR “cardioception” OR “HEP”)
**D**	Neuroimaging technique terms	(“electroencephalography” OR “electroencephalogram” OR “EEG” OR “magnetoencephalography” OR “magnetoencephalogram” OR “MEG”)

### Inclusion and exclusion criteria

Studies were included if they used adult human HEP derived from EEG or MEG. Exclusion criteria encompassed animal studies (rabbits, mice, dogs, cats, rats), children and neonatal studies, adolescent studies, foetal studies, and other studies not related to HEP (such as other event-related potentials, epilepsy studies, viruses, or protein) or inaccessible studies. Two studies using intracranial EEG (iEEG) were excluded as they did not provide enough data to support robust conclusions.

### Data collection and analysis

**Figure 2:**
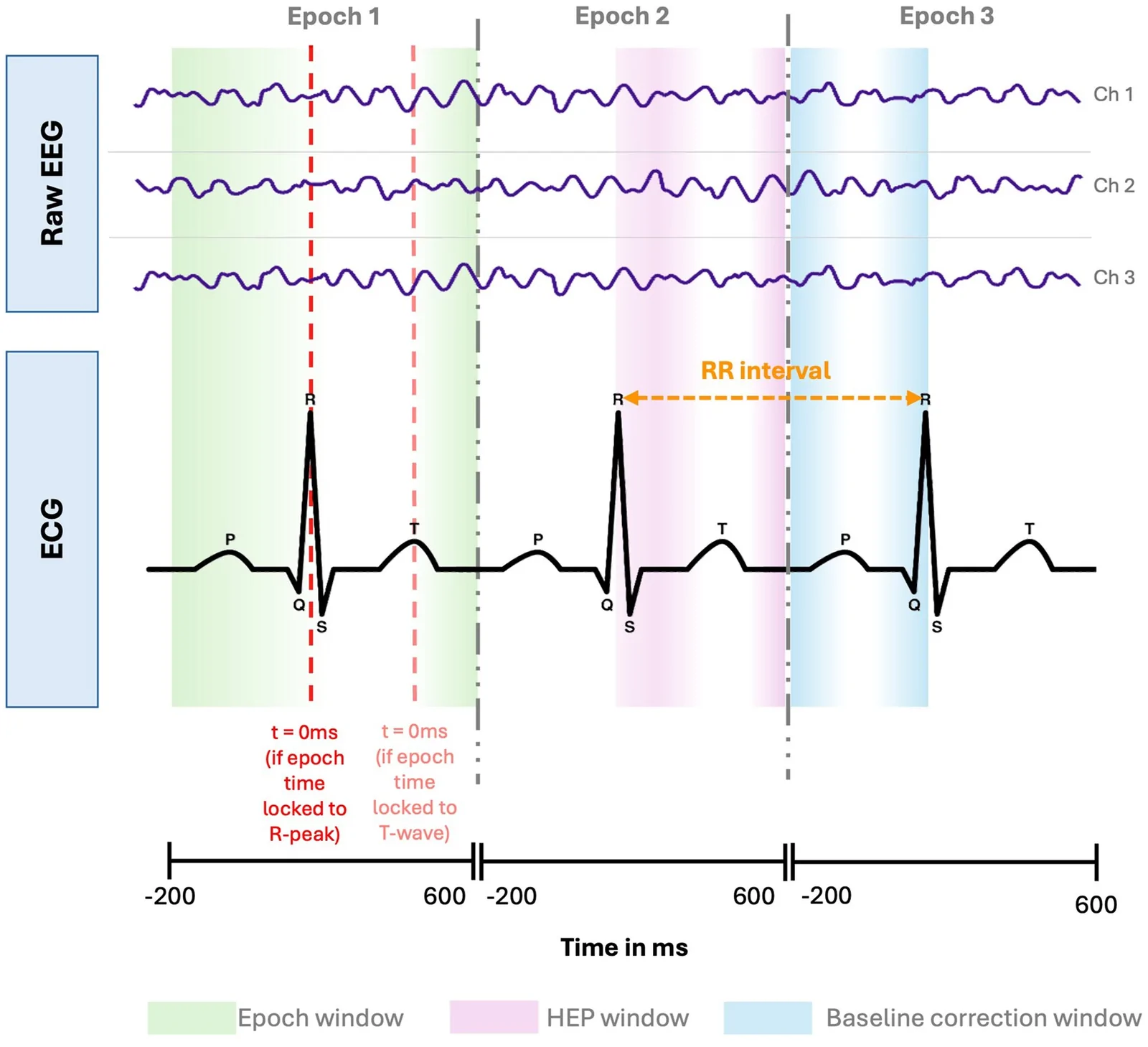
Schematic diagram of key EEG and ECG parameters used to process EEG and derive HEP. Placement of the HEP window, epoch window and baseline correction window reflects ranges identified in the scoping review and reported (see Fig. 3 and Table 5). Shading intensity indicates how frequently specific time intervals are used across studies, with darker regions denoting more commonly adopted values. The positioning of these windows varies across studies, particularly with respect to ECG landmarks. In particular, some studies define epochs spanning from the T-wave to the start of the subsequent P-wave, reflecting the idea that CFA is minimal within this interval. The use of a baseline correction remains debated by the community (Petzschner *et al*. 2019, Banellis and Cruse 2020, Verdonk *et al.* 2021), as baseline windows overlapping with early cardiac components (e.g., the P-wave) may introduce distortions in the interpretation of neural responses.

Full-text screening and data extraction was performed by the author and an independent reviewer (co-author: R.K.) flagged any inconsistencies during data extraction. Current processing techniques and parameters reported in the literature were examined and are listed below. The data extracted from the studies underwent collation, synthesis, and analysis based on the following variables and their definitions (Table 2, Fig. 2):

**Table 2:** Variables extracted from the literature and their definitions. Independent component analysis (ICA) is a signal processing technique which separates EEG signal into independent source components. Some of these components may correspond to artefacts such as muscle activity or eye movements. Artefact components are then identified, removed manually and computationally. Artefact subspace reconstruction (ASR) requires an initial calibration using segments (detected manually or automatically) of relatively clean baseline EEG data. ASR is designed to handle non-stationary/non-repeating artefacts using a sliding window and principal component analysis to identify and remove high-amplitude signal components. The three Hjorth parameters—activity (variance of the signal), mobility (mean frequency of the power spectrum), and complexity (estimation of the bandwidth of the signal)—are calculated using time-domain signal processing approaches, which offer a statistical analysis of the signal’s attributes. These parameters are used to identify and remove artefacts.

Variable	Definition
Low-pass and high-pass cut-off frequencies (in Hz)	Artefact filter settings for initial bandpass processing. A low-pass filter allows low-frequency signals and blocks high-frequency signals, while a high-pass filter does the opposite.
Epoch timeframes in milliseconds (ms) (also referred to as epoch window)	Epochs are segments of data of equal lengths extracted from continuous EEG data.
Epoch relative to R wave or T wave	Epochs are typically centred around stimulus events or responses; here either to the R wave or T wave of the ECG.
Epoch amplitude rejection in microvolts (µV)	The scalp EEG amplitude recorded in any channel surpasses a predetermined threshold set in the study, resulting in the rejection of the epoch from further analysis because it is considered to be artifact.
HEP window in milliseconds (ms)	Latency range within which HEP is thought to be found—this varies between studies. Computation of the HEP at a specific lead involves computing the average baseline-corrected amplitude in this interval.
Baseline correction time frame in milliseconds (ms)	The baseline correction time frame in EEG is the time-period before stimulus onset. It serves to correct for any fluctuations or drifts in the EEG signal that could arise during recording, including movements, electrode drift, or alterations in brain activity.
Minimum RR interval in milliseconds (ms)	This is the shortest time-period between successive heartbeats that is considered for HEP analysis. The epochs associated with shorter RR intervals are excluded from analysis. The goal of this is to reduce the likelihood of the next heartbeat’s associated electrical activity from interfering with the period of EEG used to derive the HEP.
Cardiac Field Artefact (CFA) removal methodology	Methodology used to remove cardiac field artifact such as independent component analysis (ICA), artefact subspace reconstruction (ASR), or Hjorth method.
Electrode used for EEG	These are the scalp electrodes used for EEG recording.

A. Low-pass and high-pass filter cut-off frequencies in Hertz (Hz)
B. Epoch timeframes in seconds (ms)
C. Epoch relative to R wave or T wave
D. Epoch amplitude rejection in microvolts (µV)
  E. HEP window in milliseconds (ms)
F. Baseline correction time frame in milliseconds (ms)
G. Minimum RR interval in milliseconds (ms)
H. CFA removal methodology
I. Electrodes used for EEG analysis

As only three studies used the T wave for epoch centring, this parameter was not further investigated. Following data extraction, data visualization was performed in Python (version 3.13.7), using the pandas library (version 2.3.2) for data extraction and the matplotlib library (version 3.10.5) for figure generation.

## Results

### Literature scoping review—gaps and inconsistencies in HEP processing methods

Analysis of the HEP literature using the variables detailed previously, demonstrated a high level of heterogeneity in terms of the measures reported when deriving HEP in both EEG and MEG studies (Tables 3 and 4). These results highlighted the lack of consistency in reporting variables used for HEP derivation. The variables the least reported in published EEG studies were the epoch amplitude rejection threshold (72%), the number of samples (epochs) used for analysis (79%) and the minimum RR interval (86%) (Table 3). Similar results were noted for MEG results where 87.5% studies did not report on the epoch amplitude rejection value, and the minimum RR interval (62.5%) (Table 4). This variability is further highlighted by lack of terminological consensus, with the HEP referred to by at least 10 distinct terms across the literature. These include: heartbeat-related brain potentials, heartbeat evoked response (HER), heart action evoked potential, heartbeat evoked cortical responses, heartbeat-evoked potentials (HBEP), heart action-related EEG potentials, heart-evoked potentials, heartbeat-evoked brain potentials, heartbeat event related potential, heartbeat contingent scalp potentials, and R-peak locked EEG evoked responses. Notably, inconsistency extends even to hyphenation; for example, terms such as “heartbeat-evoked” and “heartbeat evoked” are used interchangeably across studies.

**Table 3:** The number of studies and the corresponding percentage where variables were unreported based on the 199 extracted EEG studies (R and T wave studies included).

Unreported variables	Number of studies	Percentage (%)
Low-pass and/or high-pass filters	33	17
Epoch window	10	5
Epochs relative to the R wave or T wave	5	3
Epoch amplitude rejection threshold	144	72
HEP windows	32	16
Number of samples used for analysis	157	79
Baseline correction	84	42
Minimum RR interval	172	86
CFA correction	90	45
Electrodes used unreported	27	14

**Table 4:** The number of studies and the corresponding percentage where variables were unreported based on the 8 extracted MEG studies.

Unreported variables	Number of studies	Percentage (%)
Low-pass and/or high-pass filters	1	12.5
Epoch window	1	12.5
Epochs relative to the R wave or T wave	0	0
Epoch amplitude rejection threshold	7	87.5
HEP windows	2	25
Number of samples used for analysis	2	25
Baseline correction	5	62.5
Minimum RR interval	5	62.5
CFA correction	3	37.5
Electrodes used unreported	2	25

### Literature scoping review—heterogeneity in HEP parameter values

**Figure 3:**
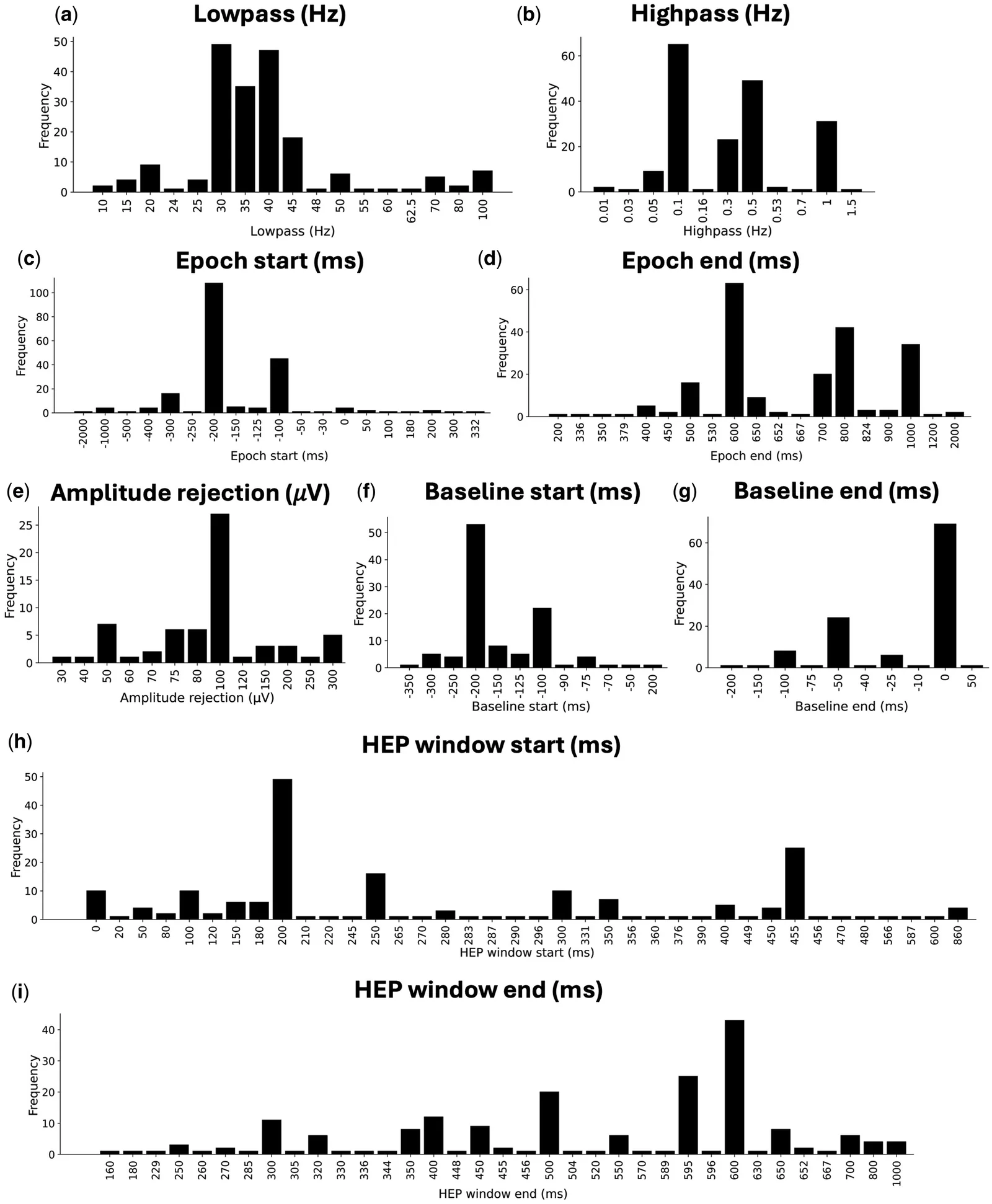
Values obtained from the literature used for each parameter (low pass (a) and high pass (b) filter cut-off frequencies, epoch start time (c) and end time (d), epoch amplitude rejection (e), baseline correction start time (f) and end time (g), HEP window start time (h) and end time (i)) to derive the HEP.

The literature demonstrated a significant level of heterogeneity in the values used for each parameter for HEP derivation (Fig. 3). This is further supported by the 200 unique combinations of preprocessing parameters (low-pass, high-pass, start and end time of epoch window, start and end time of HEP window, start and end time of baseline correction window, RR interval) extracted from the literature. This number is higher than the number of included HEP studies as some studies included two to three different time-windows for HEP extraction. To characterize the central tendency across studies, modal values were identified for each preprocessing variable (Table 5). Indeed, 49 studies (25%) out of 196 studies (excluding the T-wave studies), used 30 Hz as the low-pass filter cut-off frequency (A) with a range from 10 Hz to 100 Hz. The most common high-pass cut-off frequency (B) was 0.1 Hz used in 65 studies (33%). One-hundred and eight studies applied an epoch start time (C) at −200 ms and 63 studies (32%) utilized 600 ms as the epoch end time (D), in relation to the reference point utilized in the paper (R-peak). Two hundred milliseconds was identified as the modal value for the start of the HEP window (H) across 49 studies (25%). These studies showed an overall spread ranging from 0 ms to 860 ms, measured in relation to the R wave. Studies using the T wave as a reference point or those that were unspecified were excluded as these represented 3 of the total number of studies processed. Forty-three studies (22%) employed 600 ms as the end of the HEP window (I) with values starting from 160 ms to 1000 ms. Fifty-three studies (27%) used −200 ms for the start of the baseline correction window (F) with a range from −350 ms to 200 ms. For 69 studies (35%), 0 ms was the modal value for the end time of the baseline correction (G). Lastly, 26 studies (13%) used 100 µV as the epoch amplitude rejection threshold (E). The range of values spread from 30 µV to 300 µV.

**Table 5:** Modal values identified in the literature and the number of studies employing these values, based on the 196 HEP studies (excluding the T-wave studies) time-locked to R peaks.

HEP variable	Modal value	Number of studies	Percentage (%)
Low-pass filter	30 Hz	49	25
High-pass filter	0.1 Hz	65	33
Epoch window start time	−200 ms	108	55
Epoch window end time	600 ms	63	32
Start of the HEP window	200 ms	49	25
End of the HEP window	600 ms	43	22
Start of baseline correction window	−200 ms	53	27
End of baseline correction window	0 ms	69	35
Epoch amplitude rejection threshold	100 µV	26	13

Although a formal meta-analysis of HEP amplitudes falls outside the scope of this review—given that the substantial heterogeneity in methods reported here renders direct comparisons potentially misleading—a brief descriptive overview of HEP amplitude ranges could be informative. Across the 36 EEG studies that reported mean HEP amplitudes, values ranged approximately between −3.7 µV to +1.64 µV. Within-study standard deviations were frequently as large or larger than the reported mean HEP amplitude, highlighting the inherently low signal-to-noise ratio of the HEP. Polarity of reported amplitudes varied across studies. This reflects differences in analysis window, reference electrode, baseline correction, and scalp region of interest. All the following parameters are highly variable as reported in this review.

## Discussion

Results from the literature scoping review demonstrated a significant level of heterogeneity in the values used for HEP derivation and lack of consistency in terms of variables reported in studies (Tables 3 and 4, Fig. 3). This demonstrates the fundamental need for a processing pipeline to derive HEP to ensure replicability and consensus in this growing field.

The RR interval represents the interval in which HEP is found and comprises of one heartbeat. The minimum RR is the smallest permitted heartbeat interval in which the experimenter expects to be able to reliably find the HEP, free from interference from the subsequent heartbeat. If RR intervals are too short, the HEP extracted is more likely contaminated by the CFA, which can be an order of magnitude greater than the HEP signal. Given the importance of the RR interval, it is concerning that 86% of EEG studies do not report either whether a minimum RR interval has been used and, if so, at what value. The RR interval should be carefully considered during EEG analysis. It is important to note that the absence of RR interval reporting in earlier HEP studies likely reflects an evolving understanding of its importance rather than methodological negligence. As the HEP literature has expanded and methodological understanding has matured, awareness has grown that short RR intervals—those occurring during tachycardia for example—increase the risk of overlap between successive cardiac cycles and CFA contamination of the HEP window. This highlights a broader consideration in the field: methodological best practices are continuously updated as mechanistic understanding improves, and consensus recommendations must therefore remain dynamic rather than prescriptive.

The start and end of the HEP window are equally critical for deriving HEP and minimizing contamination by the CFA. As demonstrated by the scoping review, about 16% of studies do not report on this value, despite how critical it is to HEP analysis. Investigation of HEP windows revealed that the majority of studies (77%) use a late window, from 200 ms post-R-peak, to extract HEP. Only 8% of studies extract HEP in an earlier window (50–200 ms), suggesting that early HEP components are largely underexplored in the literature. Only 5 studies analysed multiple windows corresponding to distinct HEP components. The literature suggests that the early HEP component (100–250 ms) could reflect primary cardiac signal integration within interoceptive regions such as the amygdala and insula (Park *et al*. 2018, Gautier *et al*. 2025). The later HEP component (250–500 ms) is thought to be a non-phase locked evoked response and could reflect task-dependent processing of cardiac information (Coll *et al*. 2021, Gautier *et al*. 2025). Studies focusing exclusively on one HEP window could be failing to capture distinct physiological processes, further limiting interpretability, reproducibility and cross-study comparability. Future work should consider analysing both the early and late HEP windows and report findings accordingly.

Similarly, approximately 42% of studies do not explicitly state the baseline correction window, and approximately 45% do not report whether CFA correction was performed. Although only three studies time-locked HEP to the T-wave, precluding a meaningful methodological comparison with R-peak based approaches, this represents a valid approach with distinct implications for both HEP components captured and CFA profile. HEP components are not uniformly distributed relative to cardiac events: some components occur before the T-wave (early HEP) and some after (late HEP) (Park *et al*. 2018, Gautier *et al*. 2025), meaning that the choice of time-locking event determines which neural responses are captured. The majority of HEP studies use the same cardiac landmark (R-peak or T-wave) for both epoch time-locking and for the HEP window of interest. This is typically the R-peak. Studies should explicitly state the choice made, as the chosen landmark determines both which HEP components are expressed and the CFA profile represented in the analysis window (Park and Blanke 2019, Steinfath *et al*. 2026).

There is a fundamental trade-off between the two landmarks. The R-peak is a sharp, high-amplitude deflection, permitting reliable and consistent detection by automated algorithms and making it the methodological standard as well as facilitating study comparisons. However, using the R-peak will anchor the epoch to the point of maximum cardiac activity—systole—meaning that the early HEP window overlaps substantially with CFA. Conversely, time-locking to the T-wave and analysing HEP in the post T-wave diastolic interval leverages the period of minimal cardiac activity, possibly allowing for a cleaner separation of HEP from CFA. The trade-off is one of signal quality versus detection reliability: the T-wave is a slower, lower-amplitude, rounded waveform whose peak is inherently less well-defined and harder to systematically detect. This introduces greater variability in epoch alignment across trials and participants and may offset the advantage of reduced CFA. Future methodological work directly comparing these approaches in terms of HEP signal quality and CFA residual would be of considerable value to the field.

These results highlight the lack of consistency within the field. Hence, particular care should be given to these variables when acquiring and deriving HEP to ensure consistency and reproducibility of analysis. Based on the findings of this scoping review, several tentative recommendations can be made. These recommendations reflect current understanding and should evolve as the field progresses.

Filter selection warrants careful consideration in HEP extraction. The modal values (Table 5) of 30 Hz for the lowpass filter and 0.1 Hz for the highpass filter are broadly consistent with the current EEG practice for slow cortical potentials (Tanner *et al*. 2015). However, filter settings can affect HEP results and if not well considered can introduce distortions, spurious or attenuated amplitude effects and latency shifts. These issues are well documented in the literature (Tanner *et al*. 2015, Widmann *et al*. 2015, Luck and Gaspelin 2017). Given that HEP occupies the same low-frequency range most vulnerable to these distortions, the filter choice should be informed by empirical evaluation of their effect on HEP morphology rather than by convention alone. The community is encouraged to evaluate filter effects on their data and to report filter parameters transparently to allow meaningful study comparisons and replications.

For epoch length, windows should be sufficiently long to capture the full HEP while remaining shorter than the minimum RR interval as discussed. This is to prevent overlap with the subsequent heartbeat and CFA contamination. Particular care should be given to this for individuals with tachycardia or an elevated heart rate. The modal epoch window spanning −200 ms to +600 ms post R peak is suitable for typical resting heart rates but should be adjusted if heart rate varies substantially. Another practical approach to managing RR intervals is to apply a threshold-based epoch rejection strategy, whereby epochs below a defined minimum RR interval are excluded from analysis. The minimum RR interval used should always be reported.

Baseline correction should be applied with care as windows overlapping with early cardiac components, such as the P-wave, could introduce distortions that bias the HEP signal and compromise its interpretation (Petzschner *et al*. 2019, Banellis and Cruse 2020). If baseline correction is omitted due to possible contamination by CFA components, this should be explicitly reported. If baseline correction is applied, this should be carefully selected in order to minimize CFA overlap.

Reference electrode choices and electrodes used to derive HEP influence HEP topography and contribute to variability in the literature (Candia-Rivera *et al*. 2021, Coll *et al*. 2021). Topographic differences in reported HEP distributions in the literature may in part be reflecting reference-related decisions rather than underlying neural differences. The reference method used should always be reported and its impact on results should be considered when deriving HEP.

CFA is one of the most challenging and important steps during HEP extraction. ICA remains the most widely used approach for CFA correction. This broadly involves decomposing the EEG signal into independent components and removing those identified as artefact including CFA (Onton *et al*. 2006). However, research is showing its limitations for reliable CFA removal and its possible role in subtracting HEP with CFA (Petzschner *et al*. 2019, Buot *et al*. 2021, Abolfathi and Mohebbi 2024). Regression-based approaches model and subtract the average CFA waveform, preserving more neural signal, but assume stationarity of the CFA (Shinagawa *et al*. 2025). Other studies select a later HEP window considered to be a CFA-free zone (Antonacci *et al*. 2022, Barà *et al*. 2023). Crucially, the absence of CFA correction has been shown to introduce bias in HEP estimates. No current method perfectly separates HEP from CFA, and the choice of correction method can systematically influence HEP amplitude and topography. Whether CFA correction is applied or not, this should be explicitly reported.

The minimum number of trials required for reliable HEP extraction remains empirically unspecified and no consensus threshold currently exists. The number of trials used is also inconsistently reported in the literature. Too few trials risk yielding unreliable HEP estimates. This represents an important gap, and future work should aim to establish an empirical guideline on the minimum number of trials required for HEP extraction.

Importantly, the modal values reported in this review capture what has commonly been done in the literature, not necessarily what should be done. We do not recommend using modal values for HEP extraction. Ultimately, a standardized HEP pipeline should be grounded in the mechanistic understanding of how each parameter affects signal quality and validity. Parameters used for HEP extraction should be reported in publications, emphasizing the need for standardized methods to enhance study comparison and reproducibility and establish a gold standard in the field.

## Conclusion

The present review indicates both heterogeneity of methods used in HEP analysis and variability in the reporting thereof. There is a clear inconsistency in the use of parameters to derive HEP as well as reporting these in the literature. Consequently, the level of reproducibility and reported results appears to be variable. However, it is hard to understand the effect these parameters have on HEP in terms of direction and magnitude. Values for HEP extraction should be reported in publications, to enhance study comparison and reproducibility and eventually establish a gold standard in the field. Pipelines used to derive HEP should be made available on repositories such as GitHub. While there currently is no “ground truth” for HEP, this review has aimed to investigate processing parameters that are essential to HEP extraction. Ultimately, to develop robust methods for HEP derivation, it is essential to establish a “ground truth” for HEP with a clear consensus on CFA removal. Such standardization would provide a reliable foundation for advancing research and enhancing the precision and accuracy of HEP. This review serves as a basis for further work in this matter.

## Supplementary Material

nsag057_Supplementary_Data

## Data Availability

No new data were generated in this review. All data discussed are available within the cited publications.
